# Antimicrobial Resistance Profiles of Adherent Invasive *Escherichia coli* Show Increased Resistance to β-Lactams

**DOI:** 10.3390/antibiotics9050251

**Published:** 2020-05-13

**Authors:** Margarita Martinez-Medina, Francesco Strozzi, Belén Ruiz Del Castillo, Natalia Serrano-Morillas, Nuria Ferrer Bustins, Luis Martínez-Martínez

**Affiliations:** 1 Microbiology of Intestinal Disease Group, Biology Department, University of Girona, 17003 Girona, Spain; natalia.serrano@udg.edu (N.S.-M.); nuriaa.ferrer@gmail.com (N.F.B.); 2 Data Science Departement, Enterome Biosciences S.A., 75011 Paris, France; Francescostrozzi@tecnoparco.org; 3 Service of Microbiology, Hospital Universitario Marqués de Valdecilla, Instituto de Investigación Marqués de Valdecilla (IDIVAL), 39008 Santander, Spain; biolbelen@hotmail.com; 4 Unit of Microbiology, University Hospital Reina Sofia, 14004 Córdoba, Spain; luis.martinez.martinez.sspa@juntadeandalucia.es; 5 Maimonides Biomedical Research Institute, 14004 Córdoba, Spain; 6 Department of Agricultural Chemistry and Microbiology, University of Córdoba, 14004 Córdoba, Spain

**Keywords:** adherent invasive *Escherichia coli*, antimicrobial resistance, Crohn’s disease, β-lactams, AmpC β-lactamase

## Abstract

The adherent invasive *Escherichia coli* (AIEC) pathotype has been associated with the aetiology of Crohn’s disease (CD). Scarce reports have shown the antimicrobial resistance (AMR) profiles of AIEC. Despite antibiotics not being recommended to treat CD, antimicrobial therapy could be useful in stratified patients, such as AIEC carriers. We examined the antimicrobial resistance profiles of AIEC strains to identify which therapies could be effective or confer a risk for such patients. Phenotypic resistance to 30 antimicrobials was tested according to CLSI standards. AIEC (*n* = 22) and non-pathogenic *E. coli* (non-AIEC) strains (*n* = 37) isolated from the gut mucosa of 31 CD patients and 18 controls were studied. De novo genome sequencing was carried out for 39 of the 59 strains, and AMR genes were searched using the DeepARG database in these genomes and 33 additional AIEC publicly available genomes. The strains isolated from CD and controls showed similar phenotypic AMR profiles. The genomic analysis did not reveal an increased prevalence of AMR genes. However, AIEC strains were more frequently resistant to β-lactams than non-AIEC strains (11 AIEC (50%) and 5 non-AIEC (22%) strains were resistant to at least one β-lactam; *p* < 0.042). Two AIEC strains were resistant to expanded-spectrum cephalosporins. One strain carried a plasmid-mediated AmpC β-lactamase (CMY-69), and the other presented mutations in the promotor of the intrinsic chromosomal AmpC related to the hyperproduction of this enzyme. The rest of the strains were resistant to β-lactams not including expanded-spectrum cephalosporins. The majority carried TEM-related β-lactamases. Genomic analysis including external AIEC revealed that the gene *sul1* encoding for sulphonamide resistance was more frequent in AIEC strains than non-AIEC strains (34.6% vs. 9.5%, *p* = 0.030). AMR in AIEC is a matter of concern regarding the putative implication of the pathotype in CD. The high proportion of AIEC resistant to β-lactams warrants caution about the risk there may be in the use of these antimicrobials in AIEC-colonized CD patients.

## 1. Introduction

The adherent invasive *Escherichia coli* (AIEC) pathotype was described in 1999 when it was discovered in association with Crohn’s disease, a chronic inflammatory bowel disease [1]. Since then, this pathotype has been associated with Crohn’s disease by several independent groups [2,3,4,5,6,7,8,9]. Increased evidence indicates that AIEC may also be related to other intestinal disorders, such as ulcerative colitis and colorectal cancer, which have increasing incidence in industrialized countries [7,9,10,11,12].

The AIEC pathotype is defined by its ability to adhere to and invade intestinal epithelial cells, as well as its capacity to survive and replicate within macrophages while inducing the secretion of tumour necrosis factor α (TNF-α) [13]. AIEC strains are genetically diverse, belong to different phylogroups, and can carry several groups of virulence genes that are characteristic of extraintestinal pathogenic *E. coli* (ExPEC) [6]. The virulence properties of AIEC described to date can explain several features of the pathophysiology of Crohn’s disease, such as inflammation, mucosal translocation, and granuloma formation [14].

Since AIEC and other pathogens have been repeatedly associated with the aetiology of Crohn’s disease, antibiotics could be a logical approach to therapy, especially those that are effective intracellularly. The therapies that are currently applied to inflammatory bowel disease are mainly directed at the suppression of the immune system. They involve anti-inflammatory drugs (infliximab and adalimumab), corticosteroids (prednisone, methyl-prednisone, and budesonide), immunosuppressant drugs (azathioprine and mercaptopurine), biological therapy (TNF-α inhibitors), faecal transplantation or nutritional interventions, and surgery [15,16]. A systematic review suggests that particular antibiotic therapies could be useful to induce and maintain remission (e.g., clarithromycin and/or rifampin), to treat perianal fistulas (ciprofloxacin), and to prevent postoperative recurrence in Crohn’s disease (ornidazole) [17]. However, it is recommended that antibiotic therapy be used in conjunction with common maintenance therapies such as immunosuppressive drugs or biologics.

Few studies have reported on the antimicrobial resistance of AIEC. Dogan et al. [5] studied the antimicrobial resistance to 17 antimicrobials of 13 AIEC and 25 non-AIEC strains isolated from Crohn’s disease patients and controls, and used a microarray to test the carriage of 33 genes encoding resistance to β-lactams and class 1 integrons. Subramanian et al. [18] assayed the antimicrobial resistance to eight antimicrobials of eight mucosa-associated *E. coli* isolates from Crohn’s disease patients and six from controls. Taking into account the clinical relevance of AIEC in Crohn’s disease, it is valuable to have a better knowledge of the antimicrobial resistance in further AIEC collections and to gain insights about the molecular mechanisms of resistance.

In this work, we examined the antimicrobial resistance profiles of 49 *E. coli* strains that were isolated from Crohn’s disease patients and control subjects to 30 antimicrobials, which could be useful in the design of future clinical trials for the disease. We have also determined the putative mechanisms of resistance of the considered strains in vitro and in silico and compared them with the antimicrobial resistance genes present in other AIEC genomes in public databases.

## 2. Results

### 2.1. Susceptibility Profiles in AIEC and Non-AIEC Strains

Detailed antimicrobial resistance profiles of the strains are presented in Table 1, and the frequency of AIEC and non-AIEC strains resistant to each antimicrobial is shown in Table 2. The main result is that AIEC strains were more frequently resistant to β-lactams than non-AIEC strains (*p* = 0.042). In particular, 50% of AIEC strains were resistant to penicillins such as ampicillin and ticarcillin, and between 18.2% and 31.8% of them were also resistant to penicillins combined with inhibitors of β-lactamases. Moreover, 18.2% of AIEC strains were resistant to first- and second-generation cephalosporins, and about 10% were resistant to expanded-spectrum cephalosporins. However, none of the non-AIEC strains were resistant to those cephalosporins. Moreover, a higher frequency of strains resistant to cefoxitin was also detected in the AIEC collection, although the result did not reach statistical significance.

The proportion of strains resistant to other antimicrobials such as tetracyclines, aminoglycosides, and quinolones was similar between AIEC and non-AIEC strains (Table 2). Moreover, 30% of the strains were resistant to streptomycin, but none of them were resistant to other tested aminoglycosides, such as amikacin or gentamicin, and, with the exception of one strain, kanamycin. The number of resistances to antibiotics was higher in AIEC strains (mean ± SD, median, range: 4.14 ± 1.00, 2.5, 0–14) than in non-AIEC strains (2.07 ± 0.48, 1, 0–7). Furthermore, multidrug resistance (MDR) was two times more frequent among AIEC strains than non-AIEC strains (40.9% vs. 22.2%), but the result did not reach statistical significance (*p* = 0.134 and *p* = 0.136, respectively).

### 2.2. Susceptibility Profiles in E. coli From Crohn’s Disease and Control Subjects

No differences were found in the frequency of resistances between strains isolated from Crohn’s disease patients and control subjects (mean ± SD, median, range: 3.26 ± 0.72, 1, 0–14 and 2.56 ± 0.77, 1, 0–13, respectively) (Table 2) or in the proportion of MDR strains (Crohn’s disease: 35.5%, controls: 22.2%). No differences were observed in the frequency of each antibiotic tested. This contrasts with observations from classifying the strains by their pathogenic behaviour. Notwithstanding, despite no breakpoints being available for rifampin, a higher proportion of strains isolated from Crohn’s disease patients presented minimum inhibitory concentration (MIC) values >2 mg/L in comparison with those isolated from controls (71% vs. 39%, respectively, *p* = 0.029).

### 2.3. Mechanism of Resistance Associated with Strains Resistant to β-Lactams

Two patterns of antimicrobial resistance to β-lactams were found in the AIEC collection (Table 3). Eight AIEC strains presented resistance to several β-lactams not including expanded-spectrum cephalosporins (AIEC02, AIEC05, AIEC10, AIEC11, AIEC17, AIEC19, AIEC20, AIEC24, and AIEC25), and two were resistant to β-lactams including expanded-spectrum cephalosporins (AIEC08 and LF82). The rest of the strains were susceptible to all antimicrobials tested (AIEC01, AIEC04, AIEC07, AIEC09, AIEC12, AIEC14-1, AIEC15-1, and AIEC16-2) or resistant to some antimicrobials not including β-lactams (AIEC06, AIEC21, and AIEC23).

In particular, AIEC08 and LF82 exhibited resistance to ampicillin, ampicillin/clavulanic acid, ticarcillin, ticarcillin/clavulanic acid, cefazolin, cefoxitin, cefuroxime, cefotaxime, ceftazidime, cefpodoxime, cefovecin, and ceftiofur (Table 1). This antimicrobial resistance profile suggested the presence of a cephalosporinase. Phenotypic and genotypic assays to detect AmpC β-lactamases and extended-spectrum β-lactamases (ESBLs) confirmed that both strains presented either a plasmid-mediated AmpC β-lactamase (pAmpC) or a hyperproduction of chromosomal AmpC, not an ESBL. However, the polymerase chain reaction (PCR) assay for pAmpC was only positive for the AIEC08 strain, which carried the pAmpC CMY-69.

Since the genome of the strain LF82 is available in public databases [20], we could confirm the presence of a chromosomal AmpC. In comparison with the *ampC* region of *E. coli* K12, strain LF82 contains mutations at positions −73 (C to T), −32 (T to A), −28 (G to A), +17 (C to T), and the novel +23 (G to A). The T to A transversion at position −32 creates the consensus TTGTCA −35 box responsible for an increased transcription of *ampC* (Table 3), which could explain the observed resistance phenotype of LF82 [21,22,23,24].

Genomic analysis of all the strains showed that some mutations present in the promotor of the chromosomal AmpC of LF82 were also present in strains that were susceptible to β-lactams. However, not all mutations were found in a given strain, which suggests that a unique mutation is not sufficient to confer the resistant phenotype. In comparison with *E. coli* K12, no changes were observed in codons coding for amino acids 306 to 308 of the H-10 helix of AmpC, which is related to an extended-spectrum activity of this enzyme. This result is consistent with the susceptibility of LF82 to cefepime.

Genomic analysis confirmed the presence of TEM β-lactamases in the genomes of strains with resistance to non-expanded-spectrum cephalosporins except for AIEC02 and AIEC10, which did not present other β-lactamases apart from the chromosomal one (Table 3). TEM-148 was the most frequent and was present in all TEM+ strains but ECG57, which had TEM-1. Mutations previously related to AmpC hyperproduction were found in the promotor of AIEC10 (−88: C to T; −82: A to G; −18: G to A; −1: C to T; +17: T to C; +58 C to T), which could explain the resistance to ampicillin, ticarcillin, and cefuroxime.

However, these mutations were also found in susceptible strains (variant 7: AIEC07_E6, ECG04, ECG02, ECG21, ECG63, and ECG64), which raises questions about their implication in *ampC* hyperproduction. This variant was found in 6 out of 7 B1 strains, which suggests that these single-nucleotide polymorphisms (SNPs) are associated with the phylogenetic origin of the strains. The sequence of the chromosomal *ampC* promotor in AIEC02 was identical to that of strain *E. coli* K12 (Table 3). Thus, no mutations associated with AmpC hyperproduction were found, and the mechanism of resistance could not be deciphered for this strain.

### 2.4. Genomic Analysis of Antimicrobial Resistance Genes of Internal and External Strain Collections

**Figure 1:**
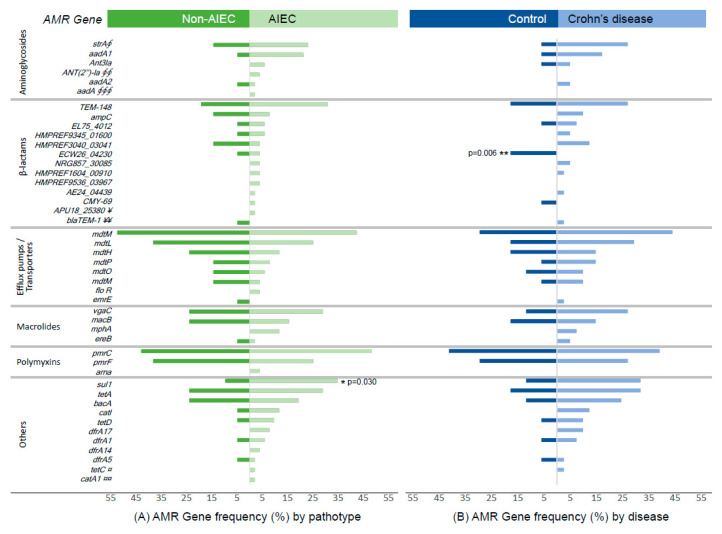
Frequency of antimicrobial resistance (AMR) genes in internal and external *E. coli* collections (**A**) by AIEC phenotype (52 AIEC and 21 non-AIEC) and (**B**) by disease origin of the strains (41 *E. coli* from Crohn’s disease and 17 from controls). To simplify the figure, AMR genes with a prevalence beyond 55% are not shown in the plot; this information can be found in Table S4. Symbols indicate additional AMR genes with the same distribution between groups than the gene indicated in the plot: § APH(6)-Id; §§ APH(3’)-Ia; §§§ AAC(6’)-Ib and AKG99_27275; ¥ HMPREF9534_03446; ¥¥ bla (ampc-EC11), ECDG_02574, and HMPREF1616_02804; ¤ BACUNI_00159; ¤¤ dfrB1.

We studied the genomic resistance profiles of internal and external strain collections to describe additional resistance genes apart from those related to β-lactams and infer the antimicrobial resistance in additional AIEC strains. The precise antimicrobial resistance (AMR) gene carriage of each strain is shown in Tables S2 and S3. Similar AMR gene distributions were found between AIEC and non-AIEC strains except for one gene (Figure 1A and Table S4). The sulfonamide resistance gene *sul1* was more prevalent in AIEC strains (AIEC: 34.62% vs. non-AIEC: 9.52%, *p* = 0.030).

The class 1 integron-integrase (*IntI1*) gene was found in 16 out of the 18 *sul1*-positive strains, suggesting that the resistance gene was present in class 1 integrons. The two other strains, AIEC25 and B2_33-1-TI5, showed partial matches with very high identity to *intI1* but corresponded only to 40–50% of the length of the *intI1* gene. Fortunately, extended-spectrum β-lactamases were not present in any other strain apart from the Spanish AIEC08 strain. However, TEM-148 was also present in the American strains MS124-1, MS85-1, MS107-1, MS79-10, and MS57-2 and the Australian strains B2-33-1-TI5, B2-52-1-TI13, B2-61-1-TI1, and B2-CD-62-LN.

A similar AMR gene distribution was also observed between strains isolated from Crohn’s disease patients and those isolated from control subjects (Figure 1B). As an exception, a chromosomal AmpC β-lactamase (beta_lactam_ECW26_04230) was more frequently found in *E. coli* isolated from controls (17.6% vs. 0%), and two genes related to aminoglycoside resistance (*strA* and *strB*) were more frequently found in *E. coli* strains isolated from Crohn’s disease patients (26.8% vs. 5.9%, *p* = 0.073).

## 3. Discussion

AIEC colonizes the intestine of 25–75% of Crohn’s disease patients [2,3,4,5,6,7,8,13], and it has been demonstrated in vitro and in vivo that they may be implicated in the pathogenesis of this disease [25,26]. Therefore, determining the antibiotic resistance profiles of AIEC may be useful for designing treatments for patients overcolonized by this pathotype or identifying therapies that could present a potential risk to patients who are colonized by resistant strains.

The most relevant result of this work is that AIEC strains were more frequently resistant to β-lactams than non-AIEC strains isolated from the intestine of Crohn’s disease and control subjects. Few studies on the antimicrobial resistance in AIEC have been published. Among them, Dogan et al. [5] showed no correlation between AIEC and antimicrobial resistance based on number or type of antimicrobial agents, including β-lactams. However, Barrios-Villa et al. [27] reported the presence of *E. coli* strains of the clone ST131 O25:H4/H30-Rx with resistance to extended-spectrum β-lactams that had AIEC virulence traits. Further studies are needed to confirm whether resistance to β-lactams is a frequent trait in other AIEC collections.

Mechanisms of resistance against β-lactams included non-extended-spectrum β-lactamases TEM-148 and TEM-1 and the extended-spectrum β-lactamase CMY-69, as well as mutations in the *ampC* promotor that could be related to increased AmpC expression. Genomic analysis of AMR gene carriage in other publicly available AIEC genomes showed a similar pattern of genetic mechanisms of resistance. These strains had at least a chromosomic *ampC* β-lactamase, and nine of them also harboured TEM-148, which corresponded to 30% of the strains studied, which is a very similar proportion to that in our strain collection (33%). Fortunately, none of the external AIEC strains had genes encoding for extended-spectrum β-lactamases. However, a recent work has demonstrated the presence of CTX-M15 and OXA-1 extended-spectrum β-lactamases in four AIEC strains isolated form healthy subjects [27].

Genomic analysis of AMR genes also revealed an increased frequency of the *sul1* gene in AIEC strains in comparison to non-AIEC strains. Sul1 is a sulphonamide-resistant dihydropteroate synthase that is normally found linked to other resistance genes located in Class 1 integrons. The *intI1* gene was found in the genomes of the majority of *sul1*-positive AIEC strains, which confirmed the presence of Class 1 integrons in these strains. Only two *sul1*+ strains were *intI1*–, but partial sequences with high identity were found. Thus, poor genome quality could have hampered the identification of the *intI1* gene in these genomes. Alternatively, the *sul1* gene could be present in the chromosome or other genetic elements that are different from class 1 integrons in these strains [28]. Class 1 integrons are often embedded in plasmids and transposons that carry several resistance genes, and are one of the main vehicles for the spread of antibiotic-resistance genes. Moreover, it has been suggested that class 1 integrons could also harbour virulence genes such as the S fimbriae subunit gene *sfaS* and siderophore receptor genes *fyuA* (ferric yersiniabactin receptor gene) and *iutA* (ferric aerobactin receptor gene) [29].

A similar proportion of resistant phenotypes was found for all the antimicrobials tested between strains isolated from Crohn’s disease patients and control subjects, with the exception of rifampin. In contrast to our observations, Dogan et al. [5] found a higher percentage of MDR strains in *E. coli* from Crohn’s disease patients than those from healthy subjects. Particularly, there was a higher frequency of strains resistant to ciprofloxacin, rifaximin/rifampin, and trimethoprim/sulfamethoxazole.

In line with our results, Subramanian et al. [18] and Eliott et al. [30] also did not find higher resistance in *E. coli* strains isolated from either Crohn’s disease patients or controls. Despite a breakpoint for rifampin not being available for *E. coli*, we found a higher proportion of strains with MIC values >2 mg/L in Crohn’s disease *E. coli* strains than in control *E. coli* strains. A non-absorbable variant of this antibiotic (rifaximin) has been commonly used in the management of inflammatory bowel disease [17]. A possible explanation for the decreased bactericidal effect of rifampin in Crohn’s disease *E. coli* strains could be the selection of resistant strains due to the previous consumption of rifampicins by such patients, but unfortunately, we do not have access to this information. In a previous study [5], rifaximin resistance was detected in 25% of *E. coli* strains isolated from ileal Crohn’s disease patients and in none of the *E. coli* isolated from healthy subjects, and strongly correlated with prior exposure of patients to rifaximin (*p* < 0.01).

Antimicrobials used for the management of Crohn’s disease have mainly been anti-mycobacterials, rifampins, metronidazole, clarithromycin, and ciprofloxacin [17]. Despite the antimicrobials tested in this work not covering all these types of antimicrobials, we provide information about the antimicrobial resistance towards rifampin and ciprofloxacin in a collection of *E. coli* strains isolated from Crohn’s disease patients and controls, and some of them have the AIEC phenotype. We found a low frequency of ciprofloxacin-resistant strains (around 4%), so this antimicrobial could be considered to treat patients overcolonized by *E. coli* or specifically AIEC.

Clinical trials in Crohn’s disease patients have demonstrated that this fluoroquinolone can be useful to treat Crohn’s disease fistulas [17]. Moreover, it has been demonstrated in vitro that rifaximin interferes with multiple steps implicated in host–AIEC interactions related to Crohn’s disease [31]. However, there is considerable risk of antimicrobial resistance to ciprofloxacin or rifaximin, especially when used empirically. Thus, non-empirical antimicrobial trials and combined therapy can be considered for patients colonized by resistant strains [5].

Clinical management of Crohn’s disease does not generally include β-lactam treatment, but this group of antibiotics is frequently used to treat many human and animal infections. However, it has been reported that the repeated use of cephalosporins and extended-spectrum penicillins during childhood was associated with increased risk of Crohn’s disease in a Finnish cohort [32]. We hypothesize that the use of penicillins could favour the selection of AIEC in the intestine, but we have no data about the antibiotic treatment history of the patients from which the *E. coli* strains were isolated to test this hypothesis.

An elegant study based on animal models demonstrated that the use of streptomycin, vancomycin, and gentamicin can promote the initial infection of AIEC and worsen infection in previously colonized hosts [33]. The exact mechanism is not clear, but it may be related to the impact of these antimicrobials on the gut microbial community, which will reduce the colonization resistance, as well as the ability of AIEC to use antibiotic-induced inflammation-derived metabolites. There are no data about the impact of using β-lactams in the expansion of AIEC in the gut.

This study provides new evidence of antimicrobial resistance in AIEC and non-AIEC strains isolated from the intestinal mucosa of Crohn’s disease and control patients and, to our knowledge, this is the first study in which a genome-based analysis has been performed to identify genetic mechanisms of antibiotic resistance in AIEC. Antimicrobial resistance in AIEC is a matter of concern regarding the putative implication of the pathotype in Crohn’s disease. Before prescribing any antimicrobial therapy for a Crohn’s disease patient, we believe that the identification of AIEC carriers and the characterization of the antimicrobial resistance profiles of AIEC from these AIEC carriers could be very relevant to preventing bad clinical outcomes for patients. Moreover, considering the impact that antimicrobial treatments have on the gut microbiota composition, even promoting the colonization and expansion of AIEC in some cases, alternative precision antimicrobial therapeutics should be further explored, such as FimH (Type 1 fimbrial adhesin) antagonists [34], colicins [35], and other strategies.

## 4. Materials and Methods

### 4.1. Strains

In a previous work, a collection of 49 *E. coli* strains were isolated from the intestinal mucosa of Crohn’s disease patients (*n* = 31 strains, 16 of which were AIEC) and control subjects (*n* = 18 strains, 6 of which were AIEC) [6]. The strains comprised 22 AIEC strains and 27 non-pathogenic clinical isolates of *E. coli* (non-AIEC). The study was approved by the ethics committee of clinical investigation of the Hospital Josep Trueta of Girona. Patients did not receive antibiotics for two months prior to colonoscopy. This strain collection is referred as “internal strains” in this study. The main characteristics of the strains are detailed in Table 1.

### 4.2. Antimicrobial Susceptibility Determination

We selected the most used antimicrobial agents in clinical and veterinary practice, including a large variety of antimicrobial categories. The isolates were tested for resistance to ampicillin, amoxicillin/clavulanic acid at a 2:1 ratio, piperacillin/tazobactam (tazobactam at a fixed concentration of 4 mg/L), cefuroxime, cefoxitin, cefotaxime, ceftazidime, cefepime, ertapenem, imipenem, amikacin, gentamicin, nalidixic acid, ciprofloxacin, tigecycline, and trimethoprim/sulfamethoxazole. The tests were conducted using the Vitek^®^2 system (bioMérieux, Madrid, Spain).

Resistance to ticarcillin, ticarcillin/clavulanic acid (fixed concentration of 2 mg/L of clavulanic acid), cefazolin, cefovecin, ceftiofur, cefpodoxime, enrofloxacin, marbofloxacin, doxycycline, chloramphenicol, and rifampin was further tested using a COMPAN1F Sensititre standard susceptibility plate (TREK Diagnostic Systems, Birches Industrial Estate, East Grinstead, United Kingdom). Isolates were also tested for resistance to tetracycline, kanamycin, and streptomycin by a macrodilution test according to the standards of the Clinical and Laboratory Standards Institute (CLSI) [36].

The MICs were interpreted according to the CLSI guidelines [37]. For the analysis of data, we considered breakpoints that fell in the intermediate and resistant categories altogether as resistant. MDR was considered when the strains were resistant to at least one agent in three or more antimicrobial categories as described previously [19].

### 4.3. Detection of AmpC β-Lactamases

Two strains presented an antimicrobial resistance profile that suggested the presence of either a plasmid-mediated AmpC-type β-lactamase or hyperproduction of the intrinsic chromosomal AmpC of *E. coli*. To test this possibility and test for the presence of an ESBL, a phenotypic disk diffusion assay on Mueller Hinton agar plates was performed with or without 250 mg/L of cloxacillin and discs of cefotaxime, ceftazidime, and cefepime with and without clavulanic acid in all three cases. Cloxacillin has an inhibitory effect on AmpC, so the test was considered positive if the diameters of the inhibition zones were ≥5 mm in the plates with cloxacillin [38]. Molecular detection of pAmpC was also performed by PCR as described previously [39].

### 4.4. Genome Sequencing of AIEC Strains

Thirty-nine strains (18 AIEC and 21 non-AIEC) out of the 49 strains analysed phenotypically for antimicrobial susceptibility were sequenced. Genome assemblies were generated starting from Illumina MiSeq (San Diego, CA, USA) raw sequencing reads (2 × 300 bp). Overlapping read pairs were stitched using FLASH v1.2.6 software with the following options: —max-mismatch-density = 0.25 —min-overlap = 10 —max-overlap = 300. De novo assembly of the stitched reads was performed with CLC-Assembly Cell 4.2.2 (Qiagen, Aarhus, Denmark) with default parameters.

### 4.5. Genomic Analysis for the Detection of Antimicrobial Resistance Genes and ampC Variants

In total, 72 *E. coli* genomes were analysed, including the genomes of the 18 AIEC and the 21 non-AIEC strains that were internally sequenced, as well as 33 additional AIEC genomes that were retrieved from public databases (external AIEC strains) [20,40,41,42,43,44,45] (Table S1). The contig sequences of the 72 genomes and 3 plasmids (from LF82, NRG857c, and UM146 strains) were processed with Prodigal v2.6.3 (Oak Ridge National Laboratory, Oak Ridge, TN, USA) [46] in single-genome mode to predict open reading frames (ORFs). The nucleotide sequences of the predicted genes were analysed with the deepARG pipeline [47] to identify antibiotic resistance genes. DeepARG (Virginia Tech, Blacksburg, VA, USA) is an in-silico analysis pipeline that uses deep learning to characterize and annotate antibiotic resistance genes in bacterial genomes and metagenomes. The pipeline compares input gene sequences with information available from a database of reference antibiotic resistance genes, using a prediction model to properly asses sequence similarities and predict if a gene can actually confer resistance to certain types of antibiotics. The deepARG pipeline (version August 2018) was run in long sequences mode (—genes) with a probability cut-off setting of 0.9 and identity cut-off of 80%. The provided DeepARG-DB was used as a reference for curated antibiotic resistance gene sequences.

All the genes annotated as members of the β-lactamase class of resistance were isolated, and the ones corresponding to the *ampC* genes were analysed separately. For the three plasmids, no *ampC* gene sequence was identified. In total, the nucleotide sequences of *ampC* genes for 72 samples, including 200 bp upstream of the starting codon, were retained and aligned using Muscle v3.8.31 (https://www.drive5.com/muscle/) [48] with default parameters. The multiple sequence alignments in Fasta format were processed with a Python v3.6.4 script, and BioPython v1.72 (https://biopython.org/) [49] was used to extract the nucleotides present in all the non-consensus positions for each of the 72 samples. The positions were reported relative to the starting codon of the K12 *E. coli* strain, which was set as the zero coordinate. The lists of antibiotic resistance genes and nucleotide variations on the *ampC* genes for each sample were compiled into two separate tables using Pandas v0.23.3 (https://pandas.pydata.org/).

For the Class I integrase gene search, the *intI1* gene sequence from a representative *E. coli* strain (O104:H4) was downloaded from the Ensembl Bacteria database (Release 45, http://eg45-bacteria.ensembl.org/). The *intI1* gene sequence was searched in the predicted genes for the 72 strains and the 3 plasmids using BlastN (v2.7.1+, NCBI, Bethesda, MD, USA) with the following parameters: -word_size 11 -perc_identity 95. The alignment results were further filtered to retain only results where at least 90% of the *intI1* representative gene sequence was aligned on one of the genes for a particular strain. The analysis pipelines and workflows were executed using Nextflow (SeqEra Labs, Barcelona, Spain) [50].

### 4.6. Statistics

Differences in the frequency of resistant strains between groups (AIEC vs. non-AIEC and Crohn’s disease vs. controls) were tested with Pearson X^2^ or Fisher exact tests (when counts within each group were ≥5 or <5, respectively). Quantitative data were compared by the Mann–Whitney U test. Statistical analyses were performed using SPSS IBM Statistics 25 15.0 software (Armonk, NY, USA).

## Figures and Tables

**Table 1:** Characteristics of the strains and resistance profiles to the antimicrobials tested. All strains were susceptible to cefepime, ertapenem, imipenem, amikacin, gentamicin, and tigecycline, so these antimicrobial agents are not included in the table. AIEC: adherent invasive *Escherichia coli*; MIC: minimum inhibitory concentration; C: control subjects; CD: Crohn’s disease; I: intermediate; R: resistant; S: susceptible.

Strain	Pathotype	Phylogroup	Disease	Ampicillin	Ticarcillin	Amoxicillin-clavulanate	Piperacillin-tazobactam	Ticarcillin-clavulanate	Cefazolin	Cefuroxime	Cefotaxime	Ceftazidime	Cefpodoxime	Cefovecin	Ceftiofur	Cefoxitin	Tetracycline	Doxycycline	Kanamycin	Streptomycin	Nalidixic acid	Ciprofloxacin	Enrofloxacin	Marbofloxacin	TMP/SMX	Chloramphenicol	Rifampin^§^
AIEC10	AIEC	A	C	R	R	S	S	S	S	R	S	S	S	S	S	S	S	S	S	S	S	S	S	S	S	S	>2
AIEC19	AIEC	A	C	R	R	S	S	S	S	S	S	S	S	S	S	S	R	I	S	R	S	S	S	S	S	S	>2
AIEC23	AIEC	A	CD	S	S	S	S	S	S	S	S	S	S	S	S	S	R	R	S	S	S	S	S	S	S	S	>2
AIEC24	AIEC	A	CD	R	R	R	R	R	I	S	S	S	S	S	S	S	R	R	S	S	R	R	R	R	R	R	>2
AIEC07	AIEC	B1	C	S	S	S	S	S	S	S	S	S	S	S	S	S	S	S	S	R	S	S	S	S	S	S	>2
AIEC04	AIEC	B2	C	S	S	S	S	S	S	S	S	S	S	S	S	S	S	S	S	S	S	S	S	S	S	S	2
AIEC06	AIEC	B2	C	S	S	S	S	S	S	S	S	S	S	S	S	S	R	R	S	I	S	S	S	S	S	S	<1
AIEC08	AIEC	B2	C	R	I	R	S	I	I	R	R	R	R	R	R	R	S	S	S	I	S	S	S	S	S	S	2
AIEC01	AIEC	B2	CD	S	S	S	S	S	S	S	S	S	S	S	S	S	S	S	S	S	S	S	S	S	S	S	>2
AIEC02	AIEC	B2	CD	R	R	R	S	I	S	S	S	S	S	S	S	S	S	S	S	S	S	S	S	S	S	R	>2
AIEC05	AIEC	B2	CD	R	R	S	R	S	S	S	S	S	S	S	S	S	R	S	S	S	S	S	S	S	S	S	2
AIEC09	AIEC	B2	CD	S	S	S	S	S	S	S	S	S	S	S	S	S	S	S	S	S	S	S	S	S	S	S	>2
AIEC11	AIEC	B2	CD	R	R	R	R	I	I	R	S	S	S	S	S	S	R	R	S	R	S	S	S	S	R	S	>2
AIEC12	AIEC	B2	CD	S	S	S	S	S	S	S	S	S	S	S	S	S	S	S	S	S	S	S	S	S	S	S	>2
AIEC14-1	AIEC	B2	CD	S	S	S	S	S	S	S	S	S	S	S	S	S	S	S	S	R	S	S	S	S	S	S	>2
AIEC15-1	AIEC	B2	CD	S	S	S	S	S	S	S	S	S	S	S	S	S	S	S	S	S	S	S	S	S	S	S	>2
AIEC16-2	AIEC	B2	CD	S	S	S	S	S	S	S	S	S	S	S	S	S	S	S	S	R	S	S	S	S	S	S	>2
AIEC21	AIEC	B2	CD	S	S	S	S	S	S	S	S	S	S	S	S	S	S	S	S	R	S	S	S	S	S	S	>2
AIEC25	AIEC	B2	CD	R	R	R	S	I	S	S	S	S	S	S	S	S	R	R	S	R	S	S	S	S	S	S	>2
LF82	AIEC	B2	CD	R	R	R	R	R	R	R	R	R	R	R	I	R	S	S	S	S	S	S	S	S	S	S	>2
AIEC17	AIEC	D	CD	R	R	S	S	S	S	S	S	S	S	S	S	S	S	S	S	R	S	S	S	S	S	S	2
AIEC20	AIEC	D	CD	R	R	S	S	I	S	S	S	S	S	S	S	S	S	S	S	R	S	S	S	S	S	S	2
ECG16	NON AIEC	A	C	S	S	S	S	S	S	S	S	S	S	S	S	S	S	S	S	S	S	S	S	S	R	S	2
ECG22	NON AIEC	A	C	S	S	S	S	S	S	S	S	S	S	S	S	S	S	S	S	I	S	S	S	S	S	S	2
ECG18	NON AIEC	A	CD	R	R	S	S	S	S	S	S	S	S	S	S	S	S	S	S	R	R	R	R	S	S	S	>2
ECG19	NON AIEC	A	CD	S	S	S	S	S	S	S	S	S	S	S	S	S	S	S	S	S	S	S	S	S	S	S	>2
ECG65	NON AIEC	A	CD	S	S	S	S	S	S	S	S	S	S	S	S	S	S	S	S	S	S	S	S	S	S	S	>2
ECG23	NON AIEC	At.	C	R	R	S	S	S	S	S	S	S	S	S	S	S	R	I	S	R	S	S	S	S	R	S	>2
ECG04	NON AIEC	B1	C	S	S	S	S	S	S	S	S	S	S	S	S	S	S	S	S	S	S	S	S	S	S	S	>2
ECG46	NON AIEC	B1	C	S	S	S	S	S	S	S	S	S	S	S	S	S	R	I	S	S	R	S	S	S	S	S	2
ECG02	NON AIEC	B1	CD	S	S	S	S	S	S	S	S	S	S	S	S	S	S	I	S	S	S	S	S	S	S	S	>2
ECG21	NON AIEC	B1	CD	S	S	S	S	S	S	S	S	S	S	S	S	S	R	I	S	R	S	S	S	S	S	S	>2
ECG63	NON AIEC	B1	CD	S	S	S	S	S	S	S	S	S	S	S	S	R	S	S	S	S	S	S	S	S	S	S	>2
ECG64	NON AIEC	B1	CD	S	S	S	S	S	S	S	S	S	S	S	S	S	S	S	S	S	S	S	S	S	S	S	>2
ECG08	NON AIEC	B2	C	S	S	S	S	S	S	S	S	S	S	S	S	S	S	S	S	S	S	S	S	S	S	S	2
ECG12	NON AIEC	B2	C	S	S	S	S	S	S	S	S	S	S	S	S	S	S	S	S	R	S	S	S	S	S	S	>2
ECG13	NON AIEC	B2	C	S	S	S	S	S	S	S	S	S	S	S	S	S	S	S	S	R	S	S	S	S	S	S	>2
ECG17	NON AIEC	B2	C	S	S	S	S	S	S	S	S	S	S	S	S	S	S	S	S	S	S	S	S	S	S	S	2
ECG41	NON AIEC	B2	C	R	R	R	S	I	S	S	S	S	S	S	S	S	S	S	S	I	S	S	S	S	S	S	2
ECG43	NON AIEC	B2	C	S	S	S	S	S	S	S	S	S	S	S	S	S	S	S	S	S	S	S	S	S	S	S	2
ECG49	NON AIEC	B2	C	S	S	S	S	S	S	S	S	S	S	S	S	S	R	R	I	R	S	S	S	S	S	S	2
ECG01	NON AIEC	B2	CD	R	R	R	S	I	S	S	S	S	S	S	S	S	R	R	S	R	S	S	S	S	S	S	2
ECG05	NON AIEC	B2	CD	S	S	S	S	S	S	S	S	S	S	S	S	S	S	S	S	S	S	S	S	S	S	S	2
ECG09	NON AIEC	B2	CD	R	R	S	S	S	S	S	S	S	S	S	S	S	R	I	S	R	S	S	S	S	R	R	2
ECG15	NON AIEC	B2	CD	S	S	S	S	S	S	S	S	S	S	S	S	S	S	S	S	S	S	S	S	S	S	S	2
ECG26	NON AIEC	B2	CD	S	S	S	S	S	S	S	S	S	S	S	S	S	S	S	S	S	R	S	S	S	S	S	>2
ECG42	NON AIEC	B2	CD	S	S	S	S	S	S	S	S	S	S	S	S	S	S	S	S	S	S	S	S	S	S	S	2
ECG34	NON AIEC	D	CD	S	S	S	S	S	S	S	S	S	S	S	S	S	R	I	S	S	S	S	S	S	S	S	>2
ECG57	NON AIEC	D	CD	R	R	S	S	S	S	S	S	S	S	S	S	S	R	R	S	R	S	S	S	S	R	S	2

**§** The breakpoint for rifampin is not available from NCCLS for *E. coli*, so the MIC categories are indicated in the table. At.: Atypical; TMP/SMX: Trimethoprim-sulfamethoxazole.

**Table 2:** Frequency of resistant strains within the *E. coli* collection studied. Differences in antimicrobial resistance between AIEC and non-AIEC, as well as between *E. coli* isolated from Crohn’s disease (CD-*E. coli*) and control (C-*E. coli*) subjects, were tested by Pearson X^2^ or Fisher exact tests as appropriate. Antimicrobial categories follow the indications of Magiorakos et al. [19] to further identify multidrug-resistant (MDR) strains. ns: no statistical significance.

Antimicrobial Category	Antimicrobial	All Strains (*n* = 49)	Non-AIEC (*n* = 27)	AIEC (*n* = 22)	*p*	C-*E. coli* (*n* = 18)	CD-*E. coli* (*n* = 31)	*p*
**β-lactams**								
*Penicillins*	Ampicillin	17	22.2	50	0.042	27.8	38.7	ns
	Ticarcillin	16	22.2	50	0.042	27.8	38.7	ns
*Penicillins*+ β-*lactamase inhibitors*	Amoxicillin-clavulanate	8	7.4	31.8	0.034	11.1	22.6	ns
	Piperacillin-tazobactam	4	0	18.2	0.035	0	12.9	ns
	Ticarcillin-clavulanate	9	7.4	31.8	0.034	11.1	22.6	ns
*Non expanded spectrum cephalosporins (1st and 2nd gen.)*	Cefazolin	4	0	18.2	0.035	5.6	9.7	ns
	Cefuroxime	4	0	18.2	0.035	11.1	6.5	ns
*Expanded spectrum cephalosporines (3d and 4th gen.)*	Cefotaxime	2	0	9.1	ns	5.6	3.2	ns
	Ceftazidime	2	0	9.1	ns	5.6	3.2	ns
	Cefpodoxime	2	0	9.1	ns	5.6	3.2	ns
	Cefovecin	2	0	9.1	ns	5.6	3.2	ns
	Ceftiofur	2	0	9.1	ns	5.6	3.2	ns
	Cefepime	0	0	0	ns	0	0	ns
*Cephamycins*	Cefoxitin	4	0	13.6	0.084	5.6	6.5	ns
*Carbapenems*	Ertapenem	0	0	0	ns	0	0	ns
	Imipenem	0	0	0	ns	0	0	ns
**Tetracyclines**	Tetracycline	15	29.6	31.8	ns	27.8	32.3	ns
	Doxycycline	15	33.3	27.3	ns	27.8	32.3	ns
**Aminoglycosides**	Amikacin	0	0	0	ns	0	0	ns
	Gentamicin	0	0	0	ns	0	0	ns
	Kanamycin	1	3.7	0	ns	5.6	0	ns
	Streptomycin	22	40.7	50	ns	55.6	38.7	ns
**Quinolones**	Nalidixic acid	4	11.1	4.5	ns	5.6	9.7	ns
*Fluoroquinolones*	Ciprofloxacin	2	3.7	4.5	ns	0	6.5	ns
	Enrofloxacin	2	3.7	4.5	ns	0	6.5	ns
	Marbofloxacin	1	3.7	4.5	ns	0	6.5	ns
**Folate Pathway Inhibitors**	Trimethoprim-sulfamethoxazole	6	14.8	9.1	ns	11.1	12.9	ns
**Phenicols**	Chloramphenicol	3	3.7	9.1	ns	0	9.7	ns
**Glycylcyclines**	Tigecycline	0	0	0	ns	0	0	ns

**Table 3:** Resistance profiles to β-lactams and molecular mechanisms of resistance. Resistance profiles were determined phenotypically, and mechanisms of resistance were determined by genomic analysis and in vitro. Single-nucleotide polymorphism (SNP) positions in the promotor region of chromosomic AmpC β-lactamase are indicated with respect to the start point for the *E. coli* K12 promoter. All the positions between −148 and +58 with identical nucleotides to *E. coli* K12 are not indicated. Different nucleotide variants in these sequences are indicated by a different number. nr: not resolved.

			Positions at the *ampC* Promotor with SNPs										
Strain	Resistance Profile to β-Lactams	Mechanism of Resistance	−88	−82	−73	−32	−28	−18	−1	+17	+23	+58	Variant
K12			C	A	C	T	G	G	C	C	G	C	1
LF82	expanded-spectrum cephalosporins	Mutations in *ampC* promotor	C	A	T	A	A	G	C	T	A	C	2
AIEC08	expanded-spectrum cephalosporins	BLEE (CMY-69)	-	-	-	-	-	-	C	C	G	C	nr
AIEC02	not expanded-spectrum cephalosporins	Mechanism not deciphered	C	A	C	T	G	G	C	C	G	C	1
AIEC11	not expanded-spectrum cephalosporins	TEM β-lactamase (TEM-148)	C	A	C	T	G	G	C	C	G	C	1
AIEC19	not expanded-spectrum cephalosporins	TEM β-lactamase (TEM-148)	C	A	C	T	G	G	C	C	G	C	1
AIEC20	not expanded-spectrum cephalosporins	TEM β-lactamase (TEM-148)	C	A	C	T	G	G	C	C	G	C	1
AIEC24	not expanded-spectrum cephalosporins	TEM β-lactamase (TEM-148)	C	A	C	T	G	G	C	C	G	C	1
ECG18	not expanded-spectrum cephalosporins	TEM β-lactamase (TEM-148)	C	A	C	T	G	G	C	C	G	C	1
ECG57	not expanded-spectrum cephalosporins	TEM β-lactamase (TEM-1)	C	A	C	T	G	G	C	C	G	C	1
ECG09	not expanded-spectrum cephalosporins	TEM β-lactamase (TEM-148)	C	A	T	T	A	G	C	C	G	C	3
AIEC05	not expanded-spectrum cephalosporins	TEM β-lactamase (TEM-148)	C	A	T	T	A	G	C	T	G	C	4
ECG41	not expanded-spectrum cephalosporins	TEM β-lactamase (TEM-148)	C	A	T	T	G	G	C	C	G	C	5
AIEC17_E1	not expanded-spectrum cephalosporins	TEM β-lactamase (TEM-148)	C	A	T	T	G	G	C	C	G	T	6
AIEC25	not expanded-spectrum cephalosporins	TEM β-lactamase (TEM-148)	C	A	T	T	G	G	C	C	G	T	6
ECG01	not expanded-spectrum cephalosporins	TEM β-lactamase (TEM-148)	C	A	T	T	G	G	C	C	G	T	6
AIEC10	not expanded-spectrum cephalosporins	Mutations in *ampC* promotor	T	G	C	T	G	A	T	C	G	T	7
AIEC12	not resistant to β-lactams	none	C	A	C	T	G	G	C	C	G	C	1
AIEC23	not resistant to β-lactams	none	C	A	C	T	G	G	C	C	G	C	1
ECG16	not resistant to β-lactams	none	C	A	C	T	G	G	C	C	G	C	1
ECG19	not resistant to β-lactams	none	C	A	C	T	G	G	C	C	G	C	1
ECG34	not resistant to β-lactams	none	C	A	C	T	G	G	C	C	G	C	1
ECG42	not resistant to β-lactams	none	C	A	C	T	G	G	C	C	G	C	1
ECG46	not resistant to β-lactams	none	C	A	C	T	G	G	C	C	G	C	1
ECG65	not resistant to β-lactams	none	C	A	C	T	G	G	C	C	G	C	1
ECG26	not resistant to β-lactams	none	C	A	T	T	A	G	C	T	G	C	4
ECG43	not resistant to β-lactams	none	C	A	T	T	A	G	C	T	G	C	4
AIEC01_E4	not resistant to β-lactams	none	C	A	T	T	G	G	C	C	G	C	5
AIEC04	not resistant to β-lactams	none	C	A	T	T	G	G	C	C	G	C	5
AIEC06	not resistant to β-lactams	none	C	A	T	T	G	G	C	C	G	C	5
AIEC09	not resistant to β-lactams	none	C	A	T	T	G	G	C	C	G	C	5
AIEC14_1	not resistant to β-lactams	none	C	A	T	T	G	G	C	C	G	C	5
AIEC15_1	not resistant to β-lactams	none	C	A	T	T	G	G	C	C	G	C	5
AIEC16_2	not resistant to β-lactams	none	C	A	T	T	G	G	C	C	G	C	5
AIEC21	not resistant to β-lactams	none	C	A	T	T	G	G	C	C	G	C	5
ECG17	not resistant to β-lactams	none	C	A	T	T	G	G	C	C	G	C	5
ECG05	not resistant to β-lactams	none	C	A	T	T	G	G	C	C	G	T	6
ECG15	not resistant to β-lactams	none	C	A	T	T	G	G	C	C	G	T	6
AIEC07_E6	not resistant to β-lactams	none	T	G	C	T	G	A	T	C	G	T	7
ECG02	not resistant to β-lactams	none	T	G	C	T	G	A	T	C	G	T	7
ECG04	not resistant to β-lactams	none	T	G	C	T	G	A	T	C	G	T	7
ECG21	not resistant to β-lactams	none	T	G	C	T	G	A	T	C	G	T	7
ECG63	not resistant to β-lactams	none	T	G	C	T	G	A	T	C	G	T	7
ECG64	not resistant to β-lactams	none	T	G	C	T	G	A	T	C	G	T	7

## Supplementary Materials

The following are available online at https://www.mdpi.com/2079-6382/9/5/251/s1, Table S1: Characteristics of the strains used for the genomic analysis, Table S2: Genomic analysis of AMR genes presence in AIEC strains, Table S3: Genomic analysis of AMR genes presence in non-AIEC strains, Table S4: AMR gene prevalence in strains grouped by phenotype (Non-AIEC/AIEC) and disease origin (Control/CD).
